# The Post-verbal Effect of Negators in Mongolian Contradictory Negations Provides Support for the Fusion Model

**DOI:** 10.3389/fpsyg.2021.603075

**Published:** 2021-05-20

**Authors:** Qinghong Xu, Shujun Zhang, Jie Li, Baizhou Wu, Helin Qiu, Qilimuge

**Affiliations:** ^1^The Key Laboratory of Psychology, Inner Mongolia Normal University, Hohhot, China; ^2^School of Psychology, Inner Mongolia Normal University, Hohhot, China; ^3^School of Foreign Languages, Yulin University, Yulin, China; ^4^Baotou Municipal Party School, Baotou, China; ^5^Institute of Psychology, Chinese Academy of Science, Beijing, China

**Keywords:** contradictory negation, Mongolian-Mandarin bilingual, post-position effect, fusion model, schema-plus-tag model

## Abstract

There are two contending models regarding the processing of negation: the *fusion model* and the *schema-plus-tag model*. Most previous studies have centered on negation in languages such as English and Mandarin, where negators are positioned before predicates. Mongolian, quite uniquely, is a language whose negators are post-verbal, making them natural replicas of the schema-plus-tag model. The present study aims to investigate the representation process of Mongolian contradictory negative sentences to shed light on the debate between the models, meanwhile verifying the post-verbal effect of negators. A series of experiments using the *sentence–picture verification paradigm* supports the fusion model: (i) Mongolian contradictory negative sentences were processed by representing the actual conditions rather than the negated state of affairs at 250 ISI (interstimulus interval of 250 ms), and (ii) despite the fact that a *post-verbal effect of negators* was measured at 250 ISI when Mongolian and Mandarin negative sentences were compared, Mongolian–Mandarin bilinguals adopted the same representational strategy for contradictory negation in both languages.

## Introduction

For syntactic processing, negative stand-alone sentences can be divided into two types: *contradictory negative sentences* from which syntactic features the actual conditions can be explicitly inferred and *non-contradictory negative sentences* from which syntactic features the conditions cannot be inferred. In other words, contradictory negative sentences have only one alternative state such as in *The window is not open*. Though no explicit information about the window's condition has been shown, the sentence provides enough information to generate the actual representation, that is *The window is closed*. In comparison, non-contradictory negative sentences have multiple alternative states, such as in *The skirt is not red*, where multiple representations such as *The skirt is blue/brown/yellow*, etc. are plausible.

Comprehension of negative sentence statements is cognitively more demanding than that of affirmative sentence statements, as latencies are longer and more error prone. At present, two models are discussed concerning the processing of negations: *dual-* and *single-step processing models*.

Both models differ concerning how the negation tag and the core supposition are processed. In the fusion model, they are spontaneously fused (e.g., Brewer and Lichtenstein, 1974; Horn, 1989; MacDonald and Just, 1989; Gannon and Ostrom, 1996; Lea and Mulligan, 2002; Mayo et al., 2004), and in the schema-plus-tag model, they are separately processed (e.g., Fiedler et al., 1996; Rubaltelli and Slovic, 2008). Put differently, the dual-step model assumes that a negation such as *The window is not open* is processed by the *negation-incongruent schema* and *an open window* by the core supposition. In contrast, a single-step model states that in the case of a closed window, a negation-congruent schema will be spontaneously activated. This distinction is important as both schemas are embedded in distinct cognitive networks. Both hypotheses have been supported by relevant studies, though we note a trend toward the dual-step model (Kaup and Zwaan, 2003; Giora et al., 2004; Hasson and Glucksberg, 2006; Kaup et al., 2006, 2007a,b; Li et al., 2007; Lüdtke et al., 2008).

The sentence–picture verification paradigm became accepted as a common method for negation-processing studies along with manipulation of interstimulus intervals (ISIs). It was found that transient, image-based, and highly view-specific representations mediate only identical view matches at short ISIs, and abstract, durable representations mediate all other view matches at long ISIs (Lawson and Humphreys, 1996). Response times (RT) across studies for view-invariant matches fell in the range of 300–700 ms (e.g., Shepard and Metzler, 1971; Klatzky and Stoy, 1974; Jolicoeur, 1985; Ellis and Allport, 1986; Ellis et al., 1989). Therefore, Stanfield and Zwaan (2001) used 250 ISI to separate the span a subject needed for accessing view-specific or more view-invariant object representations. They examined the hypothesis derived from perceptual symbol theories that people mentally represent the orientation of an object implied by a verbal description. Results indicated that at 250 ISI, when a picture matched the object orientation described by a sentence, the latencies were significantly faster compared to a non-matching picture. These results confirm that 250 ISI measures the initial stage of negation processing during which the first cognitive unit will be formed.

In later negation-processing studies (e.g., Kaup et al., 2006, 2007a; Gao et al., 2011, 2017; Chen et al., 2014; Cui et al., 2016), 250 ISI was used as the time a subject needed to form a representation of a sentence's core supposition (akin to view-specific representation in the picture-matching paradigm). Measuring whether the representation facilitates the judgment on negation-congruent or negation-incongruent picture stimuli that follow after 250 ISI would elucidate whether the representation fused the negation operator with the core or not at this stage, as advocated respectively, by the single- or dual-step model.

By that same logic, Kaup et al. (2006) used 750 ISI and found a congruency effect with the actual conditions for affirmations but not for negations. At 1,500 ISI, Yan and Bai (2000) found that most subjects can complete negation processing at around 1,000 ms in the sentence–picture verification paradigm, so 1,500 ISI was taken to be the final stage in negation processing. A congruency effect with the actual conditions was found for negations but not for affirmations. The results were interpreted in favor of the dual-step model. However, Gao et al. (2011) who applied all three ISI conditions to Mandarin negatives found diverging evidence that supported the fusion model.

There are further disparities in results of negation-processing studies. For example, Kaup et al. (2006, 2007a), using the sentence–picture verification paradigm to investigate negation processing, indicated that for both contradictory and non-contradictory negative sentences, negation-incongruent schemas were represented at 250 ISI and negation-congruent schemas at longer ISIs, which supports the dual-step hypothesis. However, Kaup et al. used different experimental stimuli when investigating representations at different ISIs. For instance, to investigate negation representation at 250 ISI, non-contradictory negations, such as *There was no eagle in the sky* and *There was no eagle in the nest*, were used. Such negations render corresponding negation-congruent schema inexplicit (Kaup et al., 2007b). In contrast, the experimental materials adopted to investigate the negation representation at 750 and 1,500 ISI were contradictory negations in German such as *Die/Der/Das A war nicht X/Y* (meaning: *The A was/was not X/Y*; where X/Y stood for the contradictory states of affairs of the subject A), and here, the negation-congruent schema was explicit (Kaup et al., 2006). Therefore, the study results were inconclusive (Gao et al., 2011).

Mayo et al. (2004) used both contradictory and non-contradictory negative sentences as experimental materials. Their results indicated that processing of non-contradictory negative sentences (e.g., *The skirt is not red*) converges with the dual-step model. The core supposition (*The skirt is red*) is represented at 250 ISI. Then, it is followed by the negation-congruent representation formed by attaching a negation tag to the core supposition. In contrast, the processing of contradictory negations (such as *The window is not open*) supports the fusion model, where a negation-congruent schema and *a closed window* are spontaneously activated at 250 ISI. Similarly, Ping et al. (2014) found that for Mandarin contradictory negations, the actual conditions were available at 250 ISI, while the negated state was represented for non-contradictory negations.

These findings point to the possibility that the negated state may not typically be represented when processing negations and that type of negation (contradictory or non-contradictory) is an important factor. Gao et al. (2011) focused on Mandarin contradictory negations with a sentence–picture verification paradigm. Their results indicated a congruency effect with the actual conditions was found at 250 ISI, which again supports the fusion model.

Studies have mostly focused on negations with S–V–O (subject–verb–object) or S–L–P (subject–link verb–predicate) sentences in languages such as Mandarin or English whose negators appear before verbs. Mongolian, in contrast, is one of the Altaic Mongol languages spoken in Mongolia, west of central Asia, north Siberia, provinces of northern China, and Inner Mongolia in particular, and Mongolian negations are formed with negators appearing at the end of S–O–V or S–L–P sentences as additional components (Yu, 2010).

Research on negations has placed limited focus on languages with post-verbal negators, with one exception being the study of Foroni and Semin (2013) on how comprehension of action negation in Dutch elicits inhibitory motor activity. Previous work focused on the representation of actions in language, and evidence supports that language stimuli referring to actions activate motor processes (e.g., Pulvermüller, 2004, 2005; Buccino et al., 2005; Pulvermüller et al., 2005a,b; Zwaan and Taylor, 2006; Filimon et al., 2007; Fischer and Zwaan, 2008; Hauk et al., 2008; Winkielman et al., 2008; Foroni and Semin, 2009, 2011). Do negations activate the motor system? Subjects were exposed to sentences in the affirmative and negative forms (e.g., *I am smiling* and *I am smiling not*) while the zygomatic muscle activity on the left side of the face was measured (electromyography technique: EMG). Change in activity compared to baseline was averaged over intervals of 200 ms, giving rise to five periods of 200 ms each during the time interval considered. An important extension of this work is how the comprehension of a negated action was represented. Results indicated that comprehension of negation entails a fast inhibition of the relevant muscle, effects of which started within the very first 200 ms. In other words, the negation-congruent representation had been formed by integrating the post-verbal negation operator with the core supposition within the first 200 ms. This seems to support the fusion model except that the manipulations may not be comparable with the sentence–picture verification paradigm.

Just like Dutch, the post-verbal feature of Mongolian negators makes Mongolian negations structurally comparable to the mental representation proposed by the dual-step model. Whether this feature of negators in Mongolian makes the processing of contradictory negations in Mongolian conform with the dual-step processing model—thus overturning the findings of Mayo et al. (2004) and Ping et al. (2014) that the processing of contradictory negations fits the fusion model—remains to be verified. More importantly, if post-verbal negators are integrated spontaneously with predicates to form negation-congruent schemas that represent the actual conditions at 250 ISI [despite Mongolian negations' structural equivalence to the dual-step model as suggested by the study of Foroni and Semin (2013) on Dutch], it would be a strong evidence in support of the fusion model.

The sentence–picture verification paradigm has been commonly used for the examination of processes involved in negations. To conform with previous studies' manipulations other than the post-verbal effects of negators, the sentence–picture verification paradigm was used in the present study to investigate the processing of Mongolian contradictory negations at different ISIs to investigate the post-verbal effect of negators on contradictory negation processing. In line with existing studies (Kaup et al., 2006; Gao et al., 2011; Chen et al., 2014; Cui et al., 2016), the present study defines 250 ISI after sentence reading as the initial stage of negation processing, 750 ISI as the middle stage, and 1,500 ISI as the late stage.

Experiment 1 investigates the mental representation of negations in Mandarin and Mongolian at 250 ISI. As Mongolian negations feature post-verbal negation markers, the logic of the experiment is as follows: its processing may follow a dual-step simulation of negation processing and not be completed within the initial stage as is the case with Mandarin negation (Gao et al., 2011). Thus, the predicted result of the experiment is that response time under the Mandarin sentence–picture matching condition will be significantly shorter than under the mismatching condition. RT under the Mongolian condition will be the opposite to Mandarin, which means the processing of Mongolian negations first simulates the negated state instead of the actual state, thus facilitating RT under the mismatching condition. Experiments 2 and 3 examine the mental representation of Mandarin and Mongolian negations, respectively, at 750 and 1,500 ISI. If the processing of Mongolian negations in the experiment conforms with the dual-step model assumptions, then the logic of experiment 2 and experiment 3 is as follows: if Mongolian negation processing is completed at 750 ISI, the RT under matching conditions will be significantly faster for both language conditions, and differences between the two language conditions would be insignificant, thus supporting the dual-step model for Mongolian negation and verifying the post-verbal effect of the negation marker. Experiment 3 would be a confirmation that the processing of negations is fully completed in prior stages, in that differences between the two language conditions would disappear at this stage. If experiment 2 does not show such a predicted effect, the purpose of experiment 3 is to examine further whether the processing of Mongolian negation is not completed until the late stage.

## Experiment 1: Mental Representation at the Initial Stage of Negation Processing of Mongolian–Mandarin Bilinguals

### Method

#### Participants

Participants across the three experiments in the present study are Mongolian–Mandarin bilingual college students from a university in Inner Mongolia Autonomous Region, China. Participants were brought up in Mongolian households and received Mongolian–Mandarin bilingual school education for at least 12 years starting from primary school to graduation from high school (as mandated by government regulations). Therefore, they were all fully competent in reading, writing, and comprehending both languages. The ideal choice for the present study would have been Mongolian and Mandarin monolingual participants as there could be potential influences from participants' proficiency difference between their native language and second language. However, decades of racial integration in Mainland China have dramatically reduced the number of Mongolian monolinguals. This research included therefore bilingual participants who grew up in Mongolian households and were not exposed to all-Mandarin surroundings until school age.

A total of 49 Mongolian–Mandarin bilingual college students (11 males and 38 females) aged between 18 and 24 years, from a university in Inner Mongolia Autonomous Region, China, participated in experiment 1. All subjects had normal (or corrected to normal) vision and no hearing or reading impairments. All volunteered to participate in the experiment and were paid the equivalent of $2.00 after the experiment. None of the participants in the formal experiment took part in the stimulus assessment.

#### Material Assessment

Eighteen proficient Mongolian and Mandarin bilinguals evaluated the contradictory states of multiple objects and selected 49 objects with explicit contradictory states. The rationality and familiarity of Mongolian and Mandarin affirmative/negative sentences involving the 49 objects were rated in a single rating on a seven-point scale [1 indicated completely unreasonable and unfamiliar, 7 indicated completely reasonable and familiar, 2–6 indicated consecutive increases in ratio and familiarity as per Gao et al. (2011)] by 22 proficient Mongolian–Mandarin bilinguals. A total of 44 groups of sentences with an average score above 5.0 were selected, from which eight groups were randomly chosen and transformed into 8 × 4 questions (e.g., *Is the window closed?/Is the window open?/Is the window not closed?/Is the window not open?*) in both languages to serve as filler trials.

Corresponding pictures depicting opposite states of each object (e.g., a closed window/an open window) were black and white with 314 pixels in length and width. Another group of 28 Mongolian college students were selected to evaluate the degree of congruency between sentences and pictures of the remaining 36 groups of sentences on a seven-point scale, and 32 sets of sentences with an average score above 5.0 were selected as formal experimental material. The 32 groups of sentences and their corresponding pictures comprised eight versions [2 (sentence type: affirmation/negation) × 2 (actual state of affair: e.g., open/closed window) × 2 (congruency condition: congruent/incongruent); see Table 1] of the experimental material. Results of repeated-measures analysis of variance (ANOVA) showed that the main effect of sentence type, congruent/incongruent conditions, and the interaction between them was not significant (*p* > 0.05), indicating consistent and homogenous stimulus material. Participants who took part in the stimulus control assessment were excluded from participating in the experimental trials.

**Table 1:** An example of one object's eight versions in experimental material.

**Sentence type × congruency condition**	**Sentence**		**Picture**
	**Mandarin**	**Mongolian**	
Affirmation × congruent	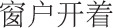 (The window is open.)	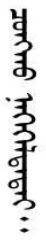	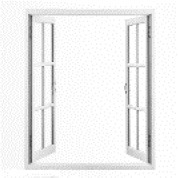
Affirmation × congruent	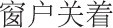 (The window is closed.)	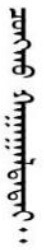	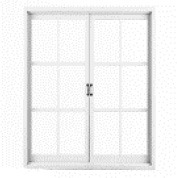
Affirmation × incongruent	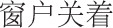 (The window is closed.)	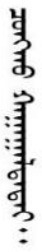	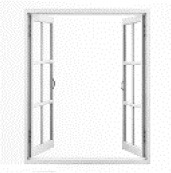
Affirmation × incongruent	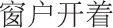 (The window is open.)	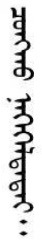	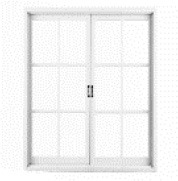
Negation × congruent	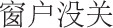 (The window is not closed.)	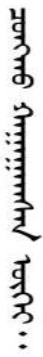	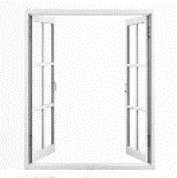
Negation × congruent	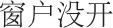 (The window is not open.)	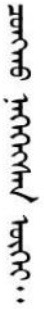	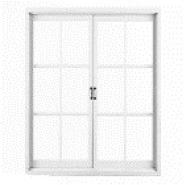
Negation × incongruent	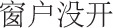 (The window is not open.)	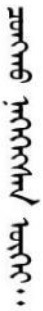	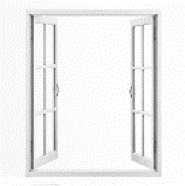
Negation × incongruent	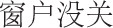 (The window is not closed.)	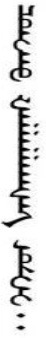	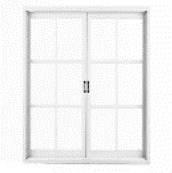

#### Stimuli

A total of 32 objects that have explicit contradictory states in Mandarin and Mongolian were used in the experiments. As for the negative sentences, the negator “not” appeared in the middle of the *subject–predicate complement* structure in Mandarin negations, that is, *subject (S)* + *not* + *predicate (X/Y)* (a linking verb is not needed here in Mandarin). For example, 
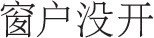
 (*the window is not open* in Mandarin), while the negator “not” appeared at the end in Mongolian negations, that is, *subject (S)* + *predicate (X/Y)* + *not* (linking verb not needed here in Mongolian), for example, “
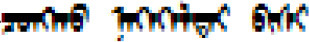
” (*the window is open not* in Mongolian). The corresponding affirmation was *subject (S)* + *predicate (X/Y)* for both Mandarin and Mongolian, e.g., 
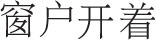
 and “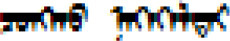.” X/Y stood for the contradictory states of the subject. There were 32 pairs of pictures in total, each pair displaying contradictory states of the same situation, such as *an open window* and *a closed window*. All images were black and white with length and width of 314 pixels.

There were 32 objects and eight versions of each, i.e., 2 (sentence type: affirmative and negative) × ss2 (sentence implied state: state X and state Y) × 2 (picture described state: state X and state Y). These were arranged by a balanced Latin square order and divided into eight sets, so that each set of experimental materials contained any one of the eight possible versions as well as 32 Mandarin sentences and 32 Mongolian sentences with the same meaning but different syntactic structures (affirmative or negative). Each participant was randomly assigned to one set.

We included fillers that had different syntactic structures to the experimental items to avoid that participants would develop strategies for parsing affirmative and negative sentences. Twenty of the 32 filler stimuli (transformed from eight randomly selected sets of sentences) with consistency ratings between sentence and pictures above 5.0 on the seven-point scale, followed by *Yes* or *No* choices, were added to the experiment. The filler stimuli were the same across the eight sets of materials and were presented in random order together with experimental stimuli.

It should be pointed out that Mandarin sentence materials were horizontally presented and Mongolian vertically, as is conventional in each language. Although the difference in printing format may cause unwanted influences, we believe such influences were minor as eye-tracking studies (e.g., Yan and Bai, 2000) have found that native Mandarin participants were able to read vertically printed sentence materials as quickly as horizontally printed texts.

#### Procedure

The experiment adopted a 2 (language: Mongolian/Mandarin) × 2 (sentence type: affirmative/negative) × 2 (sentence–picture congruency condition: congruent/incongruent) within-subjects design. The materials were presented with the sentence–picture verification paradigm. Participants would first see a sentence at the center of the screen. After reading a sentence, they were asked to press the space bar as soon as possible so that a “+” sign appeared at the central fixation point during a 250-ms ISI before a picture was presented. Participants were instructed to press the “J” key for sentence–picture congruent associations and the “F” key for incongruent associations. They were asked to do so as accurately and quickly as possible. After the response, the next set of materials would be automatically and randomly presented. The filler items included questions that required an affirmative or negative response by pressing the “J” or “F” key. Each experiment consisted of two blocks and was conducted in a balanced Latin square order, with the first half of all tasks in the order of Mandarin sentences first and then Mongolian, and the other half in a reversed order.

### Results and Discussion

The finding of Kaup et al. (2007a) was that participants responded to the questions regarding affirmative sentences with a mean accuracy of 77% and those regarding negative sentences with a mean accuracy of 80%. Thus, in the present experiment, we excluded four participants whose accuracy rates were lower than 75% (accounting for 8.2% of all data), and where RTs were longer than 3,000 ms or shorter than 300 ms (1.32%) or beyond 2 standard deviations from a participant's mean (1.76%), these were also omitted from all analysis (as per Gao et al., 2011; Cui et al., 2016). Yan and Bai (2000) noted that the average fixation duration on a Mandarin reading material was ~0.3 s for both horizontal and vertical formats, and the number of words an average participant can see at one gaze is about two (normal Mandarin printing format is horizontal, but it was found that participants were able to read vertically printed material just as quickly). In contrast, they also pointed out that the reading speed of Mandarin characters ranged from 2.9 to 11.0/s. Sentence length in the current study was mostly four characters, with slight variation in length for corresponding Mongolian sentences. Thus, 300 ms was too short for participants to respond, while 3,000 ms was too long as participants were able to process a complete sentence on average in 1.5 s (Bai et al., 2011). A statistical analysis was conducted on the valid data of 45 participants. Mean RTs and standard deviations are shown in Table 2.

**Table 2:** Means and standard deviations of response time (ms) and accuracy rate (%) at 250 ISI.

**Language**	**Congruency**	**Negation**		**Affirmation**	
		**Response time**	**Accuracy**	**Response time**	**Accuracy**
Mongolian	Incongruent	2,007 ± 689	0.82 ± 0.15	1,442 ± 438	0.92 ± 0.09
	Congruent	1,715 ± 520	0.84 ± 0.16	1,426 ± 322	0.89 ± 0.11
Mandarin	Incongruent	1,774 ± 554	0.90 ± 0.12	1,465 ± 390	0.94 ± 0.09
	Congruent	1,639 ± 486	0.84 ± 0.17	1,406 ± 377	0.92 ± 0.11

RT data were submitted to a mixed-effect model analysis, and SPSS 25.0 software was used for statistical analysis. Repeated-measures data of language type (Mongolian/Mandarin), sentence type (affirmation/negation), congruency condition (congruent/incongruent), and two-factor (language type × sentence type, language type × congruency condition, and sentence type × congruency condition) and three-factor interactions were entered into the model as fixed effects. A random subject-specific effect of treatment was then added and retained in the model. An assumption of equal correlation of residuals was plausible (within-subject design), so the compound symmetry structure was adopted for covariance structure.

**Figure 1:**
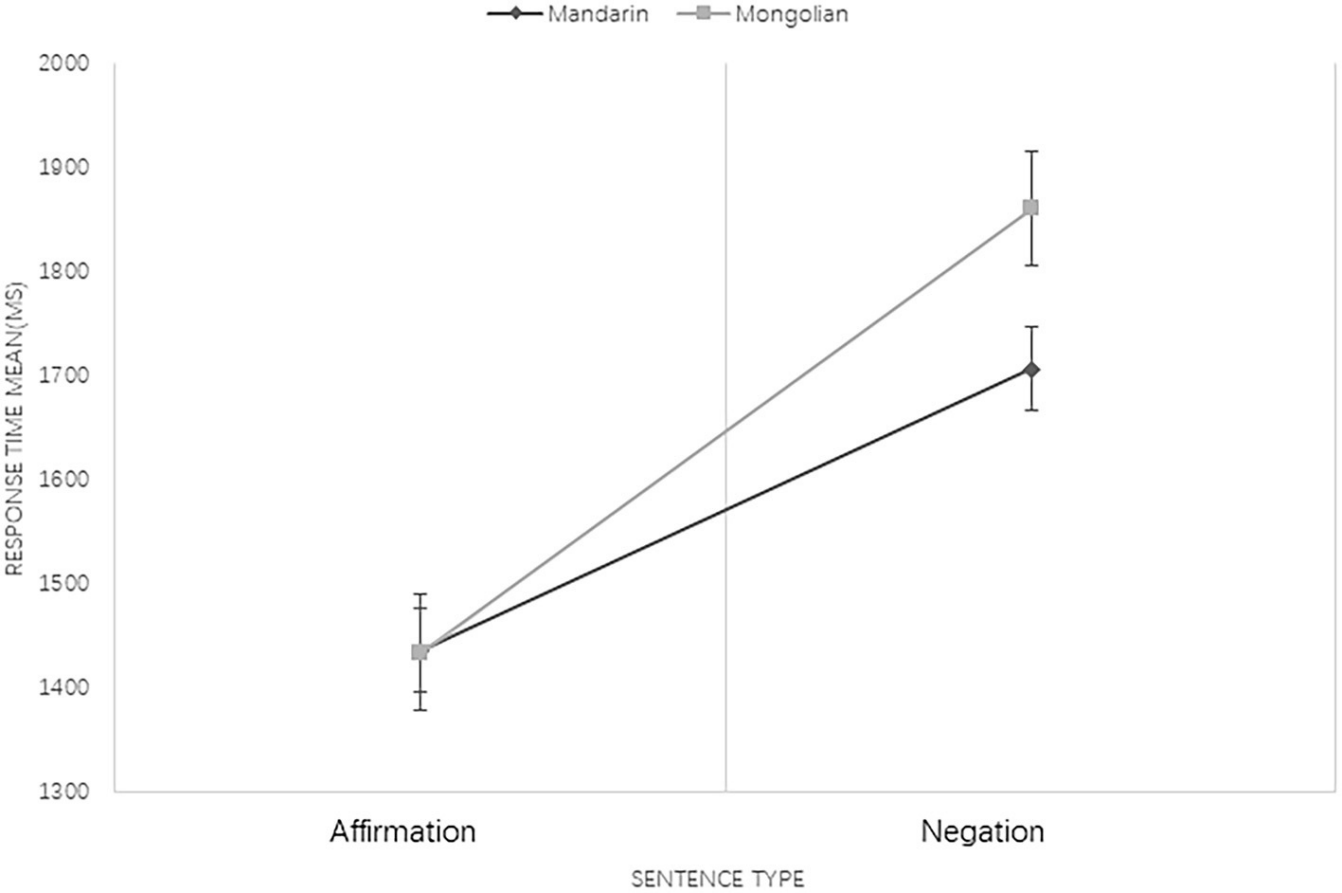
The interaction between language type and sentence type at 250 ISI. Bars represent standard error.

**Figure 2:**
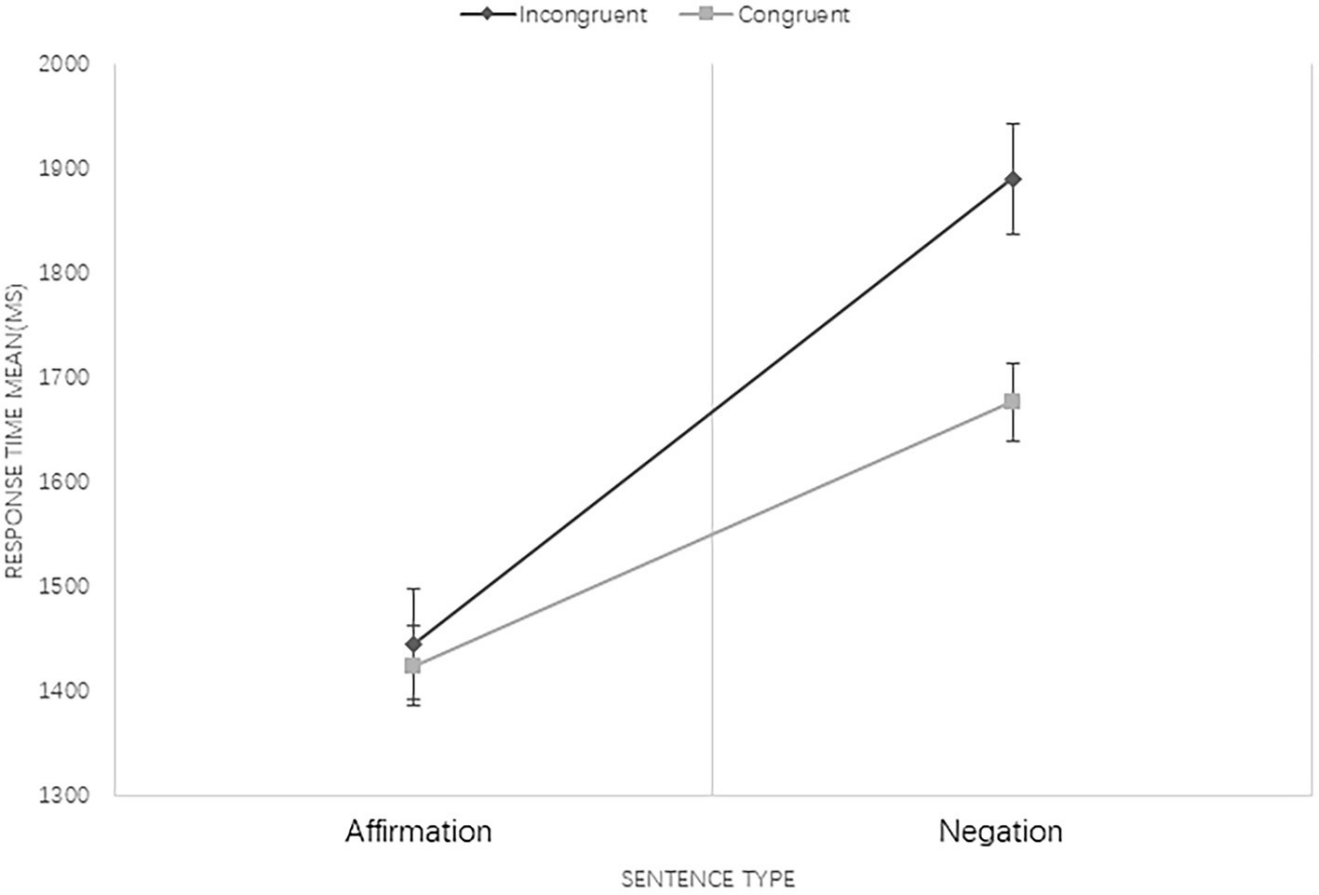
The interaction between congruency condition and sentence type at 250 ISI. Bars represent standard error.

Results revealed that the main effect of language type was not significant [*F*_(1, 308)_ = 3.727, *p* = 0.54]. The sentence type effect was significant, indicating that processing of negations took longer than affirmations [*F*_(1, 308)_ = 77.854, *p* < 0.001]. The main effect of congruency condition was significant, namely, the RT for pictures that had incongruent associations with sentences took significantly longer than congruent ones [*F*_(1, 308)_ = 8.872, *p* < 0.01]. Three-factor interaction among language type, sentence type, and sentence–picture congruency condition was not significant [*F*_(1, 308)_ = 2.166, *p* = 0.142]. The interaction of language type and sentence type was significant [*F*_(1, 308)_ = 3.892, *p* = 0.049], and a further analysis of their interaction showed that the processing of negative sentences in Mandarin was significantly faster than in Mongolian (see Figure 1). Moreover, the interaction between sentence type and congruency condition was also significant [*F*_(1, 308)_ = 5.993, *p* = 0.015]. A further analysis indicated that when processing affirmations, there was no significant difference between congruent and incongruent conditions, but RT in the congruent condition was significantly shorter than that in the incongruent condition when negations were processed (see Figure 2). The interaction between language type and congruency condition was not significant [*F*_(1, 308)_ = 0.267, *p* = 0.606].

Accuracy data submitted to logistic regression showed that the Cox–Snell *R*^2^ and Nagelkerke *R*^2^ are 0.045 and 0.086, respectively. The significance of the Hosmer–Lemeshow test is 0.683, while the effect of language type, sentence type, congruency condition, and their interactions are all insignificant. These findings indicate that the variables and their interactions exerted no significant influence on accuracy and that the possibility of a speed–accuracy trade-off can be ruled out.

Unexpectedly, the results of experiment 1 indicate that when processing contradictory negative sentences, the actual state (rather than the negated state) was represented at 250 ISI in both Mandarin and Mongolian conditions. As expected, the processing of Mongolian negations was significantly slower than that of Mandarin negations at this stage, verifying the existence of the post-verbal effect of negators in Mongolian at the initial stage of negation processing. However, such an effect did not change the fact that Mongolian negation processing does not conform with the dual-step model. To confirm that the processing of Mongolian negations is fully completed during the first stage, we carried out experiment 2 to test if the differences between both language conditions would disappear at 750 ISI.

## Experiment 2: Middle Stage Mental Representation of Negation Processing of Mongolian–Mandarin Bilinguals

### Methods

#### Participants

A total of 60 Mongolian and Mandarin bilingual college students (12 males and 48 females) aged between 18 and 23 years participated in the experiment. Participants in this experiment did not include individuals from experiment 1.

#### Stimuli

Stimuli were the same as those used in experiment 1.

#### Procedure

The same procedure was used as for experiment 1, but this time, a 750-ms ISI was applied instead of a 250-ms ISI.

### Results and Discussion

Data of seven participants whose accuracy rates were lower than 75% (accounting for 11.6% of all data) were discarded, and RTs longer than 3,000 ms or shorter than 300 ms (1.02%) as well as those beyond 2 standard deviations from a participant's mean (1.34%) were omitted. Mean RTs and standard deviations are shown in Table 3.

**Table 3:** Means and standard deviations of response time (ms) and accuracy rate (%) at 750 ISI.

**Language**	**Congruency**	**Negation**		**Affirmation**	
		**Response time**	**Accuracy**	**Response time**	**Accuracy**
Mongolian	Incongruent	2,236 ± 691	0.84 ± 0.14	1,676 ± 702	0.90 ± 0.11
	Congruent	1,922 ± 551	0.85 ± 0.12	1,649 ± 567	0.88 ± 0.11
Mandarin	Incongruent	1,982 ± 802	0.84 ± 0.12	1,591 ± 680	0.86 ± 0.14
	Congruent	1.735 ± 670	0.90 ± 0.11	1,493 ± 569	0.90 ± 0.11

**Figure 3:**
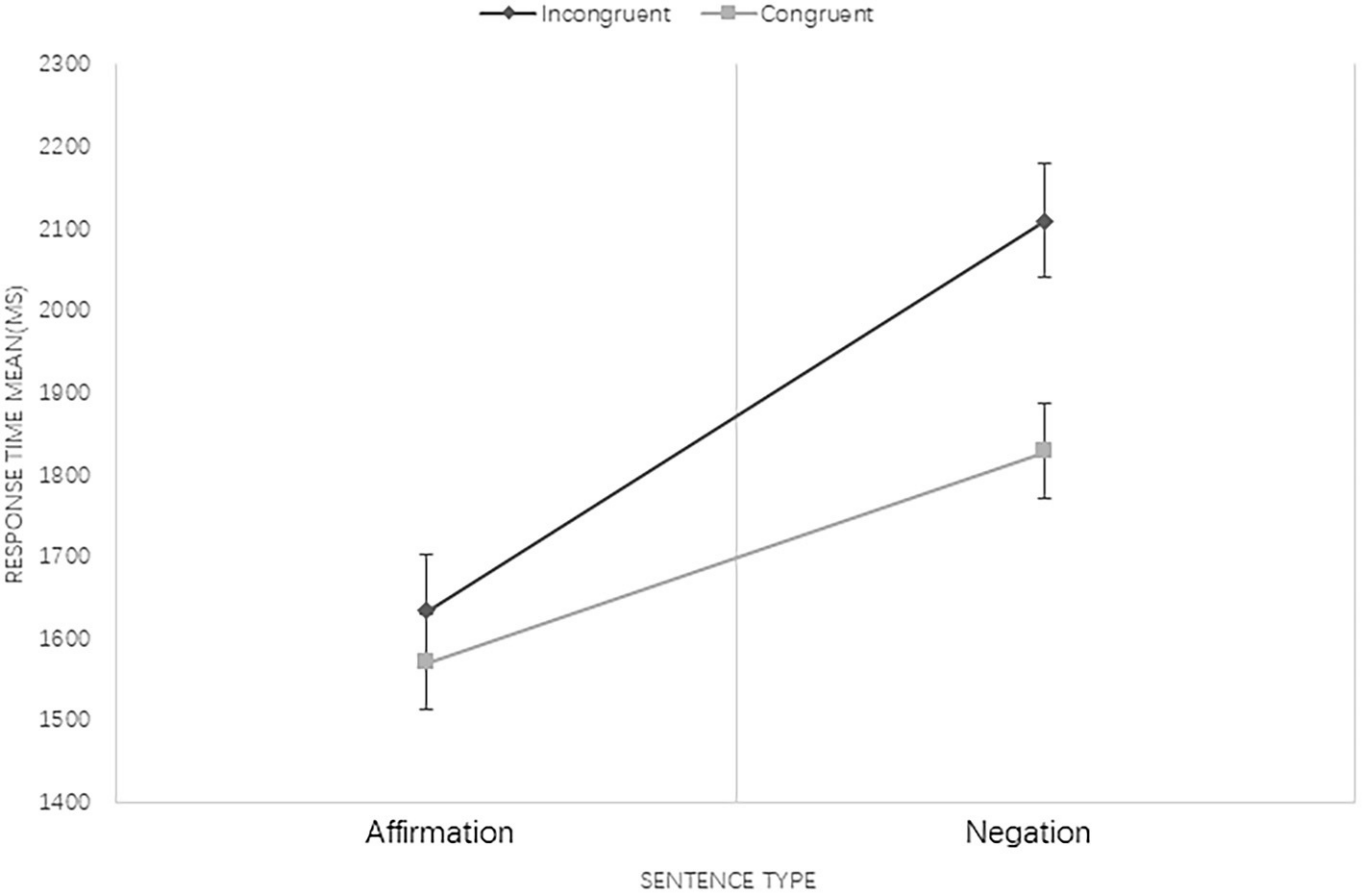
The interaction between congruency condition and sentence type at 750 ISI. Bars represent standard error.

In experiment 2, RT data were submitted to a mixed-effect model analysis with the same procedures as experiment 1. The main effect of language type was significant [*F*_(1, 336)_ = 10.147, *p* < 0.01], and the sentence type main effect was also significant, indicating that for this experiment, the processing of negations also took longer than affirmations [*F*_(1, 336)_ = 46.896, *p* < 0.001]. The main effect of congruency condition was also significant, namely, RT for pictures that were incongruent with the sentences took significantly longer than congruent ones [*F*_(1, 336)_ = 10.272, *p* = 0.001]. Neither the interaction among language type, sentence type, and sentence–picture matching state was significant [*F*_(1, 336)_ = 0.415, *p* = 0.52] nor was the interaction of language type and sentence type [*F*_(1, 336)_ = 0.870, *p* = 0.351]. The interaction of sentence types and congruency condition was significant [*F*_(1, 336)_ = 4.165, *p* < 0.05]. A further analysis revealed no significant differences between the congruent affirmative and the incongruent affirmative conditions. However, RTs in the congruent negative condition were significantly shorter than those for the incongruent negative condition (see Figure 3). The interaction between language type and congruency condition was not significant [*F*_(1, 336)_ = 0.001, *p* = 0.982].

Logistic regression of the accuracy data showed that the Cox–Snell *R*^2^ and Nagelkerke *R*^2^ are 0.022 and 0.040, respectively, and the significance of the Hosmer–Lemeshow test is 0.945. As for the first experiment, the effects of language type, sentence type, congruency condition, and their interactions are all insignificant, which is a proof again that the variables and their interactions had no significant influence on accuracy and that a speed–accuracy trade-off could be ruled out.

The results of experiment 2 suggested that the actual state (rather than the negated state) was represented at 750 ISI in both Mandarin and Mongolian. The interaction between language type and sentence type was not significant, indicating that the difference between negations and affirmations disappeared in both Mandarin and Mongolian. Therefore, we are now certain that the processing of Mongolian negations is fully completed at 250 ISI. At this point, experiment 3 seems redundant, yet Gao et al. (2011) found that when processing contradictory negative sentences in Mandarin, there was a significant effect of congruency condition at 250 ISI, which suggested that the actual situation was processed. This effect disappeared at 750 ISI but then resurfaced at 1,500 ISI. To determine whether this is also the case with Mongolian contradictory negations, we proceeded with experiment 3.

## Experiment 3: Late-Stage Mental Representation of Negation Processing of Mongolian–Mandarin Bilinguals

### Methods

#### Participants

A total of 52 Mongolian and Mandarin bilingual college students (17 males and 35 females) age between 18 and 24 years participated in the experiment. Participants in this experiment did not participate in the previous experiments.

#### Stimuli

Stimuli were the same as those used in experiment 1.

#### Procedure

The same procedure was used as for experiment 1, but this time, a 1,500-ms ISI was applied instead of a 250-ms ISI.

### Results and Discussion

Data of eight participants whose accuracy rate was lower than 75%, accounting for 15.3% of all data, were discarded. RTs longer than 3,000 ms or shorter than 300 ms (1.46%) and those beyond 2 standard deviations from the participants' mean (1.87%) were omitted. Mean RTs and standard deviations are shown in Table 4.

**Table 4:** Means and standard deviations of response time (ms) and accuracy rate (%) at 1,500 ISI.

**Language**	**Congruency**	**Negation**		**Affirmation**	
		**Response time**	**Accuracy**	**Response time**	**Accuracy**
Mongolian	Incongruent	2,267 ± 1,121	0.86 ± 0.14	1,668 ± 514	0.92 ± 0.09
	Congruent	2,067 ± 829	0.86 ± 0.14	1,649 ± 731	0.86 ± 0.12
Mandarin	Incongruent	1,958 ± 682	0.85 ± 0.13	1,589 ± 589	0.88 ± 0.10
	Congruent	1,871 ± 653	0.89 ± 0.14	1,445 ± 488	0.92 ± 0.09

In experiment 3, RT data were submitted to a mixed-effect model analysis with the same procedures as experiment 1. The main effect of language type was significant [*F*_(1, 336)_ = 6.395, *p* = 0.012] as well as the sentence type main effect, indicating that the processing of negations took longer than affirmations [*F*_(1, 336)_ = 46.847, *p* < 0.001]. The main effect of congruency condition was not significant, namely, the response time of congruent and incongruent associations was not significantly different [*F*_(1, 336)_ = 0.790, *p* = 0.375]. Furthermore, the interaction among language type, sentence type, and sentence–picture congruency condition [*F*_(1, 336)_ = 0.000, *p* = 0.987] and the interaction of language type and sentence type were not significant [*F*_(1, 336)_ = 0.002, *p* = 0.963]. Neither the interaction of sentence types and congruency condition was significant [*F*_(1, 336)_ = 0.107, *p* = 0.774] nor was the interaction between language type and congruency condition [*F*_(1, 336)_ = 1.470, *p* = 0.226].

Accuracy data submitted to logistic regression showed that the effect of language type [0.011], congruency condition [0.003], the interaction between language type and congruency condition [0.001], and the interaction between sentence type and congruency condition [0.018] are significant. However, the Cox–Snell *R*^2^ and Nagelkerke *R*^2^ are 0.025 and 0.048, respectively, and the significance of the Hosmer–Lemeshow test is 0.929, a proof that the variables and their interactions posed no significant influence on accuracy and that the possibility of a speed–accuracy trade-off could be ruled out.

The results of experiment 3 show that the actual state rather than the negated state was represented at 1,500 ISI in both Mandarin and Mongolian. The interaction between language type and sentence type was not significant, contradicting the findings of Gao et al. (2011). Their conclusion was drawn from Mandarin monolinguals. To verify that it is also applicable to Mongolian–Mandarin bilinguals, we separately analyzed the responses to the first half of tasks (which were presented in the order of Mandarin sentences first and then Mongolian) in all three experiments, as tasks presented first were not likely to be affected by initiation from a particular language. The results revealed that at 250 ISI, the congruency condition effect of both languages was significant (*M* = −258 ms, SE = 64.483, *F* = 16.008, *p* = 0.001; *M* = −337.556, SE = 92.151, *F* = 13.418, *p* = 0.002), and processing under the sentence–picture congruent condition was clearly facilitated. At 750 ISI, the congruency condition effect of Mongolian was not significant (*M* = −141.237, SE = 91.588, *F* = 2.378, *p* = 0.137), while that of Mandarin was significant (*M* = −214.747, SE = 90.122, *F* = 5.678, *p* = 0.025). At 1,500 ISI, the congruency condition effect was not significant for either language (Mongolian: *M* = −119.995 ms, SE = 88.277, *F* = 1.848; Mandarin: *M* = −122.493, SE = 65.630, *F* = 3.484). This indicates that bilinguals also integrated the core suppositions with negators within short ISIs in monolingual tasks. Bilinguals' performance was consistent with the fusion model in both language conditions at the initial stage. The suppression alternative hypothesis proposed by Giora et al. (2007) suggests that negation-congruent information in a subsequent coherent context is still active while simultaneously negation-incongruent information gets suppressed during 750–1,000 ISI. Therefore, it is plausible to conclude that the results of the present study do not support any model of the dual-step hypothesis. However, the inconsistency with the results of Gao et al. (2011) at the middle and late stages with respect to the dual-step simulation hypothesis is as follows: Gao et al. (2011) used negations with predicates that directly elicit a vicarious motor experience of the described situation from participants (e.g., *the umbrella is not opened* or *the umbrella is not closed*); contrastingly, sentence materials in the current study used an S–L–P pattern that did not emphasize explicit motor action simulations. Another possible explanation might be language proficiency difference. These interpretations call for further studies, at best with eye movement and/or brain imaging techniques.

## General Discussion

Results of experiments 1, 2, and 3 show that when processing contradictory negative sentences such as 
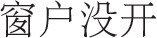
 (*the window is not open*) or “
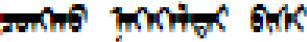
” (*the window is open not*), the main effect of congruency condition was significant in all ISI conditions. This suggests that when processing Mongolian and Mandarin contradictory negative sentences, the actual state rather than the negated state will be active even at 250 ISI, and the actual state remains active at 750 and 1,500 ISI. This is consistent with the findings on English (Mayo et al., 2004) and Mandarin contradictory negations (Ping et al., 2014); both studies support the fusion model of negation processing.

When processing a contradictory negation, the negation-congruent schema (or the actual condition) is effortlessly and spontaneously formed within a very short time span. For example, *a closed window* is represented as the negation-congruent schema of *the window is not open*, so that the actual state gets processed as an affirmative sentence of the opposite schema within 250 ms after reading the negative sentence. Our focus has been on the congruency effect of Mongolian negations at 250 ISI as it supports the fusion model. However, an alternative interpretation of the congruency effect is possible and has been brought to our attention by a reviewer of this research. The cognitive mechanisms underlying the identification of a match and a mismatch are different. A representation that is active should facilitate the response for a match and simultaneously interfere with the response for a mismatch. For instance, in *The window is not open*, a subject's ability to correctly decide that this is a mismatch is hindered by the fact that the representation of *an open window* is somewhat active. *The window is open* does not generate the representation of *a closed window*, so there is no interference in rejecting the trial when a picture of *a closed window* appears. This explains why there is no congruency effect for affirmative sentences. It suggests that the dual-step model is wrong, but that the fusion model is only partially right: the representation of the actual conditions is available from the initial stage, but the negated state is also partially available. Only further downstream (1,500 ISI) the representation of the negated state disappears as evidenced by the lack of any congruency effect.

A similar theory, the suppression/retention hypothesis proposed by Giora et al. (2007), postulated that the negated state was retained in memory instead of being thoroughly suppressed. This representation could thus facilitate relevant information in subsequent reading, so that the negated state and the actual state exist in a balanced level of activation. Kaup et al. (2006) also pointed out that in affirmative sentences, perceivers represent only *a closed door* in *The door is closed*, while in *The door is not open*, they represent both *an open door* and *a closed door*. They first focus their attention on the open door and then the closed door, and there is a certain tipping point in the comprehension process at which they shift attention away from the negated state of affairs and onto the actual condition. In conclusion, the above accounts in one way or another assume that the processing of negation involves the construction of a representation of both the negated state and the actual conditions and that perceivers somehow managed to tip the scale in favor of one over the other before the late stage of processing.

As the sentence–picture verification paradigm was not always applied identically across studies, controversies concentrated on whether the actual or the negated state gets represented at different stages as a result of different experimental manipulations leading comprehenders to adjust their “tipping point” in the representation process. For instance, Kaup et al. (2006) used sentences and pictures that pose relative positions to create different congruency conditions, e.g., *The star is not above the plus* and pictures of “^*^” and “+” are placed one above the other. Ping et al. (2014) used sentences and pictures that imply different gestures (likely to induce experiential motor simulations), e.g., *The lady is not standing* and pictures of a sitting or a standing lady. Gao et al. (2011) used experimental materials akin to ours in the current study (mostly objects with clear contradictory states, e.g., *an open/closed window*). The point is, these manipulations may lead to different “tipping points” on the part of the comprehenders. Therefore, determining whether the very same mental process was elicited in these various manipulations precedes arguing over which model is the correct one. Event-related potential (ERP) studies might be useful to address this issue in more detail. ERP components such as N400 (a component sensitive to fulfilled semantic predictions; Kutas and Hillyard, 1984; Kutas and Federmeier, 2011; Kuperberg and Jaeger, 2016) and P600 (a component characterized as a response produced by syntactic anomalies or syntactically dispreferred continuations; Osterhout and Holcomb, 1992; Hagoort et al., 1993) might prove valuable to the topic.

By comparing Mongolian and Mandarin at 250 ISI, it was shown that responses to Mongolian contradictory negative sentences were significantly slower than those to Mandarin, while there was no significant difference between Mandarin and Mongolian affirmative sentences. That is to say, it is the post-verbal negator that gave rise to the significant difference between Mongolian and Mandarin at the initial stage of negation processing. Moreover, the difference between both languages was no longer significant at longer ISIs for both negative and affirmative sentence processing. In considering an overall analysis of the three ISI conditions, it appears that the post-verbal negators lead to slower processing at 250 ISI for Mongolian, while at longer ISIs, negators were integrated with the core supposition due to the extension of processing time, as indicated by the absence of RT differences. Note that comparable RTs to congruent/ incongruent affirmations are not consistent with the results of previous studies (e.g., Kaup et al., 2006; Kaup et al., 2007a,b; Gao et al., 2011) that revealed a significant difference between RTs to congruent and incongruent pictures in the case of affirmative sentences. Two speculations were made regarding this difference: a bilingual advantage (making participants process affirmation-congruent/incongruent situations equally fast, as our study is, to our knowledge, the first one to use balanced bilingual participants) or the vertical printing of Mongolian sentences (making them process affirmation-congruent/ incongruent situations equally slowly; although studies indicate that subjects can read vertically and horizontally presented Mandarin sentences equally fast, there have been no such studies of Mongolian to date).

Linguists have already provided a plausible explanation for the post-verbal effect of negators, namely, the *distance iconic motivation*, which dictates that the linear expression of negation has its specific word order effect and that the closer the elements are in surface structure, the more they are grammaticalized or lexicalized together (Yuan, 2000). Each element in a sentence serves its unique purpose: some express presuppositions (or old information) whose meanings are maintained in negation and some contain focal meaning (or new information) that is negated in negations. Therefore, the natural sequence of expression would be pre-verbal negators that directly negate the focal meaning that closely ensues, forming a focused scope of negation. In the absence of accentuations, the scope of negation is naturally the word that closely follows the negation marker. Put differently, when processing negations, the position of negation operators affects the focus of comprehenders, because they spontaneously assume that the part following the negation marker is the focus of negation. The scope of negation is transferred by a post-verbal negator, e.g., in *The window is not open*, the adjective *open* is the only spontaneous focus of negation, and it composes the scope of negation as it comes right after the negation marker *not* in word order. However, with post-verbal negators, e.g., in Mongolian negation “
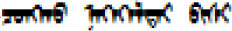
”(*The window is open not*), readers could not spontaneously assume a negation focus and, therefore, need to reread or recollect previous information to choose a focus between *window* and *open*, which are now the scope of negation (the negation remains contradictory when *open* is the focus, yet when the negation focus is *window*, the negation becomes non-contradictory in that schemas of many other things being closed could be activated, such as *a closed door* or *a closed drawer*). Usually, such negation focus choices could be assisted and facilitated by accentuations in conversations, but it is different for written language. In a word, the post-verbal negation marker adds to the complexity of negation processing by transferring and extending the scope of negation within a negative sentence, making the negation focus less clear. Combined with the findings of the present study, the fusion model receives support; although Mongolian contradictory negations have a natural structural equivalence to the dual-step model and the post-verbal of negators proves to be inefficient, Mongolian–Mandarin still successfully integrated the negator with the core supposition at 250 ISI.

This study had some limitations. Interaction between negation and congruency was found at 250 and 750 ISI but not at 1,500 ISI, which indicates that 1,500 ISI is more than enough for both sets to fully complete the integration process, thus making experiment 3 non-essential in this study. Participants' error rates were high in experiments 2 and 3, which may have resulted from some of the participants ignoring negation markers at the end of Mongolian negations despite filler trials. Moreover, the present study included only contradictory negations, which leaves it open whether the same model can be applied to non-contradictory negation processing. Finally, a discrepancy with the results of previous studies regarding RTs to congruent/incongruent affirmations remains an open question in the present study, and further studies are needed to clarify this contradiction.

To sum up, the present study went beyond the typical Western-driven research by using Mandarin and Mongolian materials and balanced bilingual participants. The fusion model, rather than the dual-step model, receives support from our findings. The negation marker and the core supposition are spontaneously integrated at 250 ISI in both languages in spite of a clear post-verbal effect of negators when Mongolian negated sentences are processed. Importantly, the findings reveal that not only the negation operator will be integrated with the core supposition but also that distance iconic motivation processing will be completed at 250 ISI.

## Data Availability Statement

The raw data supporting the conclusions of this article will be made available by the authors, without undue reservation.

## Ethics Statement

The studies involving human participants were reviewed and approved by School of Psychology Ethics Committee, Inner Mongolia Normal University. The patients/participants provided their written informed consent to participate in this study.

## Author Contributions

JL was responsible for the conception and design of the study. SZ and BW made substantial contributions to the acquisition, analysis, and interpretation of data. QX drafted the work and revised it critically for important intellectual content. HQ and Q also contributed to the experimentation and data analysis of the study. All authors contributed to the article and approved the submitted version.

## Conflict of Interest

The authors declare that the research was conducted in the absence of any commercial or financial relationships that could be construed as a potential conflict of interest.
