# Supplementary material for: The Post-verbal Effect of Negators in Mongolian Contradictory Negations Provides Support for the Fusion Model
Source: Front Psychol. 2021 May 20;12:603075. doi: 10.3389/fpsyg.2021.603075 (PMC8173153; doi:10.3389/fpsyg.2021.603075)
Supplement: Supplementary file 1 [file Data_Sheet_1.docx]

Supplementary Material

Appendix

A full list of 32 Mandarin and corresponding Mongolian sentences in one experimental material set (1 in 8).

| Mandarin | Mongolian | English Translation |
| --- | --- | --- |
| 火柴着了。  火柴没着。  火柴灭了。  火柴没灭。  灯泡亮了。  灯泡没亮。  灯泡熄灭。  灯泡没灭。  毛巾干了。  毛巾没干。  毛巾湿了。  毛巾没湿。  小狗活着。  小狗没活。  小狗死了。  小狗没死。  衣服干净。  衣服不净。  衣服脏了。  衣服不脏。  坏人跑了。  坏人没跑。  坏人停下。  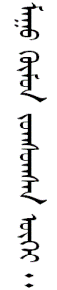坏人没停。  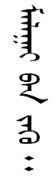  帽子戴上。  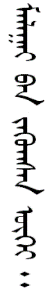  帽子没戴。  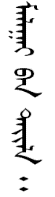  帽子摘下。  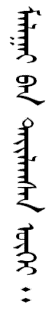  帽子没摘。  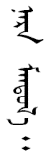太阳升起。  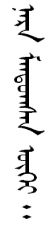  太阳没升。  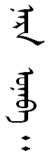  太阳落下。  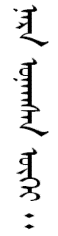  太阳没落。 | 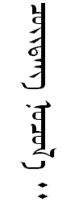  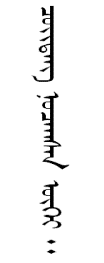  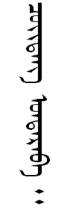  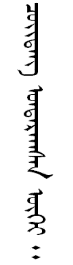  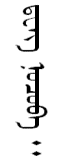  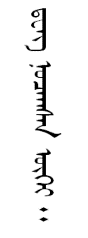  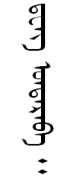  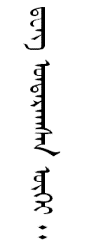  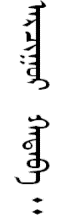  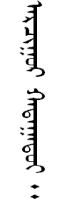  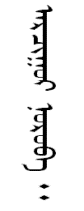  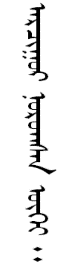  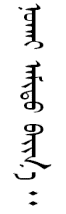  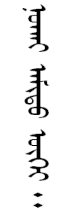  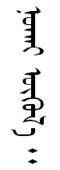  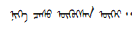  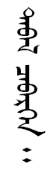  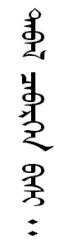  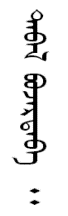  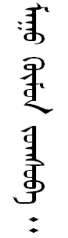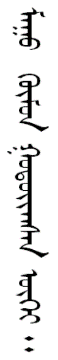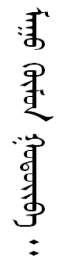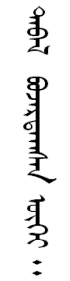 | The match is burning.  The match is not burning.  The match has burned-out.  The match has not burned-out.  The light is on.  The light is not on.  The light is off.  The light is not off.  The towel is dry.  The towel is not dry.  The towel is wet.  The towel is not wet.  The puppy is alive.  The puppy is not alive.  The puppy is dead.  The puppy is not dead.  The coat is clean.  The coat is not clean.  The coat is dirty.  The coat is not dirty.  The badman ran off.  The badman did not run off.  The badman stopped.  The badman did not stop.  Hat is on the head.  Hat is not on the head.  Hat is taken off.  Hat is not taken off.  The sun rises.  The sun didn’t rise.  The sun has set.  The sun has not set. |

A list of all 44 objects with explicit contradictory states selected as experimental material.

| Drawer | Match | Light | Towel |
| --- | --- | --- | --- |
| Puppy | Coat | Badman | Hat |
| Sun | Screen | Ball | Machine |
| Grapes | Schoolbag | Phone | Moon |
| Floor | Door | Window | Bottle cap |
| Glass | Mouth | Bag | Wings |
| Cat | Umbrella | Book | Watermelon |
| Knife | Apple | Eyes | Faucet |
| Line | Scroll | Blanket | Shoelaces |
| Shoe | Table | Bow | Clothes |
| Balloon | Tree | Cup | Egg |

Example picture


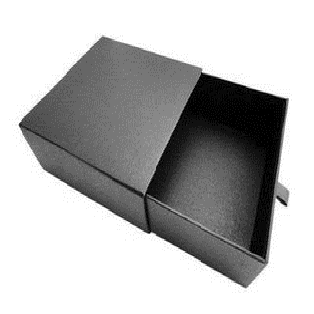

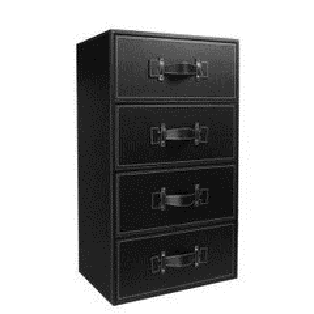


Example filler stimuli
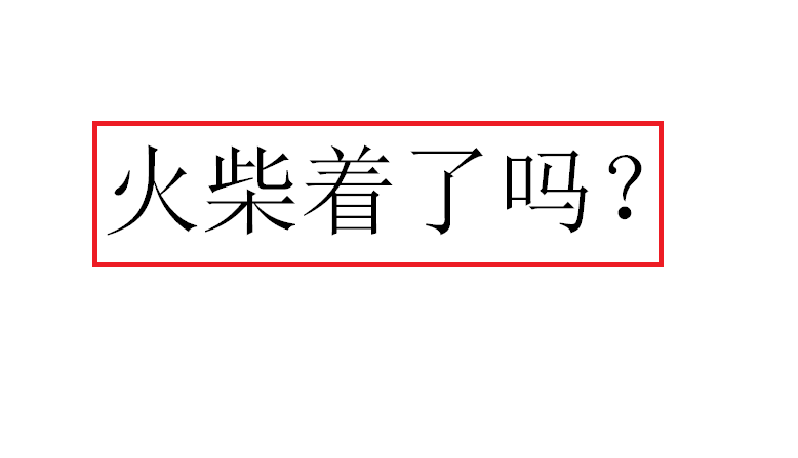

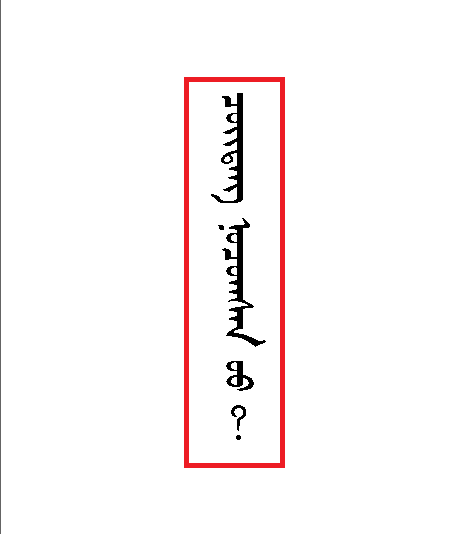


**
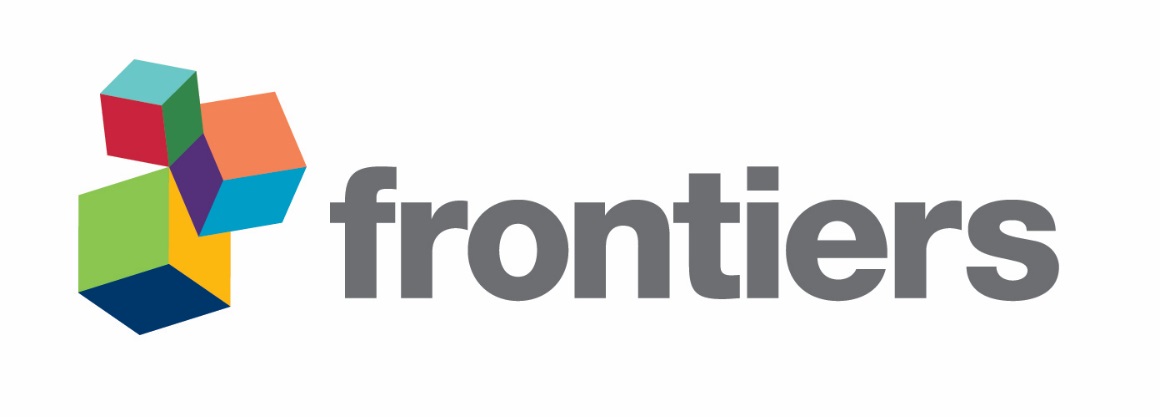
**
